# R848 Adjuvant Laden With Self-Assembled Nanoparticle-Based mRNA Vaccine Elicits Protective Immunity Against H5N1 in Mice

**DOI:** 10.3389/fimmu.2022.836274

**Published:** 2022-05-31

**Authors:** Xinyu Zhuang, Luer Chen, Songhui Yang, Shengnan Xia, Zhiqiang Xu, Tong Zhang, Boyu Zeng, Tong Yu, Ning Yu, Wei Wang, Huijun Lu, Mingyao Tian, Ningyi Jin

**Affiliations:** ^1^ Changchun Veterinary Research Institute, Chinese Academy of Agricultural Sciences, Changchun, China; ^2^ College of Veterinary Medicine, Jilin University, Changchun, China; ^3^ College of Agriculture, Yanbian University, Agricultural College of Yanbian University, Yanji, China; ^4^ College of Veterinary Medicine, Yangzhou University, Yangzhou, China; ^5^ College of Veterinary Medicine, Jilin Agricultural University, Changchun, China; ^6^ College of Animal Science and Technology, Guangxi University, Nanning, China; ^7^ Institute of Virology, Wenzhou University, Wenzhou, China

**Keywords:** mRNA vaccine, ferritin, CD5 signal peptide, R848, H5N1

## Abstract

In order to perfect the design strategy of messenger RNA (mRNA) vaccines against the H5N1 influenza virus, we investigated whether different antigen designs and the use of adjuvants could improve the immune effect of mRNA vaccines. We designed three different forms of antigen genes, including Flu [H1/H3/H5/B-HA2(aa90~105)-M2e(24aa)], Flu-Fe (Fe, ferritin), and CD5-Flu-Fe (CD5, a secretion signal peptide). Meanwhile, R848 (Requimod) was selected as the adjuvant of the mRNA vaccine. We prepared cationic lipid nanoparticles for mRNA delivery, named LNP-Man (mannose-modified lipid nanoparticles). Cell transfection results showed that Flu-Fe/CD5-Flu-Fe containing ferritin could express the target antigens HA2 and M2e more efficiently than Flu. In the mice immune experiment, five immune groups (LNP-Man/Flu, LNP-Man/Flu-Fe, LNP-Man/CD5-Flu-Fe, LNP-Man/Flu-Fe+R848, and LNP-Man/CD5-Flu-Fe+R848) and two control groups (LNP-Man, PBS) were set up. After being infected with the 1×LD_50_ H5N1 avian influenza virus, the survival rate of the mice in the LNP-Man/CD5-Flu-Fe, LNP-Man/Flu-Fe+R848, and LNP-Man/CD5-Flu-Fe+R848 were 100%. More importantly, in LNP-Man/Flu-Fe+R848 and LNP-Man/CD5-Flu-Fe+R848 groups, there was no residual virus detected in the mice lung tissue on the 5th day postchallenge. Overall, this study provides a new idea for the design of H5N1 avian influenza virus mRNA vaccines in terms of antigen designs and adjuvant selection.

## Introduction

The influenza A virus (IAV) belongs to Orthomyxoviridae, whose genome consists of eight RNA fragments of negative polarity (1). Compared with other viruses, a well-known feature of IAV is that they can be quickly adapted to new hosts through two different mechanisms: antigenic drift, which is the slow accumulation of point mutations in individual genes, and antigen shift, which is the reassortment of genomic fragments between two (or more) strains within a co-infected cell (2, 3).

In 1997, the first case of a human infected with the H5N1 subtype highly pathogenic avian influenza was reported in Hong Kong, China. Since 2003, the cumulative number of laboratory-confirmed human cases of H5N1 infection from multiple countries has been 862, including 455 deaths (4–6). Although the effective transmission of H5N1 virus from human to human remains to be further observed and studied, several previous studies have shown that humans are faced with an increasing risk of large-scale epidemics caused by the H5N1 virus due to high mortality as well as the great potential for mutation and adaptation to other hosts (7).

Ever since the US Food and Drug Administration approved the emergency use authorization of two kinds of mRNA vaccines against SARS-CoV-2 (8), mRNA-based technology has attracted wide attention from the scientific community to investors (9). Compared with the DNA vaccine, subunit vaccine, inactivated vaccine, and attenuated live vaccine (10), the mRNA vaccine is a non-infectious and non-integrated platform, so there is no potential risk of infection or insertion mutation. At the same time, mRNA is degraded through normal cellular processes, whose half-life *in vivo* can be regulated by various nucleic acid modification (11, 12) and delivery methods (13, 14).

It is generally assumed that the progress in the structural biology, nanotechnology, and gene delivery of influenza viruses can provide new opportunities for developing improved vaccines with broader protective immunity against multiple influenza viruses. In a recent innovation, several natural proteins have shown a good ability to form nanoparticles suitable for antigen presentation and immune stimulation. One of the proteins is ferritin (15), which is a ubiquitous iron storage protein self-assembled into nanoparticles (16). Masaru Kanekiyo (17) confirmed that the ferritin structure–based, self-assembling synthetic nanoparticle vaccine improves the potency and breadth of influenza virus immunity.

There are many signal peptides used to mediate the secretory expression of exogenous genes in eukaryotic cells, such as the human CD5 signal peptide (18, 19), growth hormone signal peptide, and *Saccharomyces cerevisiae* α factor signal peptide. The human CD5 signal peptide is considered to be an ideal signal peptide sequence for mediating a secretory expression of proteins (20, 21).

R848 (Requimod) is an immunomodulator that is well known for its potential antiviral activity. Clinical studies have shown that R848 can be successfully used in the treatment of infectious viral diseases, such as the hepatitis C virus or herpes simplex virus. The antiviral activity of R848 was manifested as reducing viral shedding and viral reoccurrence (22). R848 is recognized by toll-like receptors TLR7 and TLR8, which promote immune cell activation and induce a T_h1_-type immune response. In addition, R848 activates plasmacytoid dendritic cells, monocytes, and macrophages; secretes cytokines; and mediates innate and acquired immunity. When it comes to mouse studies, R848 was proven to be a vaccine adjuvant to promote an adaptive immune response (23).

In this study, we investigated whether different antigen designs and the combination of adjuvants could improve the immune effect of mRNA vaccines. We designed three different forms of antigens against the H5N1 subtype avian influenza virus, in which ferritin and CD5 signal peptide sequences were selectively introduced. Meanwhile, R848 was selected as the adjuvant of the mRNA vaccine. Finally, the mRNA vaccines were encapsulated with mannose-modified lipid nanoparticles and the immunogenicity and protective effect against the 1×LD_50_ H5N1 virus challenge in mice were evaluated.

## Materials and Methods

### Cells, Mice, and Influenza Virus

A549 (human non-small cell lung cancer cells) were cultured at 37°C in Ham’s F 12 nutrient medium (F-12) supplemented with 10% fetal bovine serum (FBS). A/chicken/Jilin/9/2004(H5N1) virus (GenBank: AY653193.1) used in this study were preserved by our laboratory. Specific pathogen-free female C57BL/6 mice were purchased from Vital River Laboratory Animal Technology Co., Ltd, Beijing, China.

All procedures involved in the animal study were performed in an animal bio-safety level 3 (ABSL-3) facility.

### mRNA Production

According to the protocol of T7-FlashScribe Transcription Kit (CELLSCRIPT, Madison, WI, USA), mRNAs were produced by T7 RNA polymerase based on linearized plasmids pGEM-3Zf-n3 (24) encoding EGFP, luciferase (Luc), codon-optimized Flu [H1/H3/H5/B-HA2(aa90~105)-M2e(24aa)], Flu-ferritin (ferritin, GenBank-M12937.1), and CD5-Flu-ferritin (CD5, GenBank-AK292698). Then, the mRNA was capped by the ScriptCap Cap-1 Capping System (CELLSCRIPT, WI, United States) and purified by the MEGAclear Transcription Clean-Up Kit (Thermo Fisher Scientific, Waltham, MA, United States). In consequence, the resulting mRNA contained the optimal Cap-1 structure. Uridines were replaced with modified N1-methyl-pseudouridine (TriLink, NorthPark, CA, United States) to dampen innate immune sensing and increase mRNA translation efficiency *in vivo*. The purified mRNA was stored frozen at −80°C until further use.

### LNP-Man/mRNA Preparation and Characterization

mRNA was encapsulated in LNP-Man (mannose-modified lipid nanoparticles, DOTAP : DOPE : DSPE-PEG-Mannose_(mol/mol)_=50:50:1) upon a self-assembly process in which an aqueous solution of mRNA was rapidly mixed with a solution of lipids dissolved in ethanol by a microfluidic device. Syringe pumps were used to perfuse the ethanol and aqueous phases at a ratio of 3:1. Formulations were concentrated using 10 kD ultra-centrifugal filters (Millipore, Billerica, MA, United States). DOTAP and DOPE were purchased from A.V.T. Pharmaceutical Co., Ltd (Shanghai, China), and DSPE-PEG-Mannose was purchased from Xi′an Ruixi Biological Technology Co., Ltd. LNP-Man/mRNA were prepared at the indicated N:P molar ratio (N, nitrogen on DOTAP; P, phosphate on mRNA). A mean molar mass of 330 Da per nucleotide was assumed for calculating the molar ratio between DOTAP and RNA, and then the particle size and zeta potential were measured according to Malvern Zetasizer Nano ZS90 (Malvern Panalytical, Westborough, MA, United States). Finally, the form was observed by transmission electron microscopy (TEM) and scanning electron microscopy (SEM).

### mRNA Transfection

A549 cells were plated at 10^6^ cells per well in a 12-well plate in Opti-MEM media, to which the prepared LNP-Man/mRNAs (mRNA = 1 μg/well) were added. After 4 h of transfection, Opti-MEM media was replaced by F-12 media containing 10% FBS. After 24 h transfection, the relating flu genes and EGFP expression were respectively monitored by Western blot and fluorescence microscopy.

For LNP-Man/mLuc transfection, A549 cells were plated at 10^4^ cells per well in a 96-well white plate in Opti-MEM media, to which the prepared LNP-Man/mLucs (mRNA = 1 μg/well) were added. After transfection, luciferase activity was determined by a microplate reader.

### Cytotoxicity Assay

The cytotoxicity of LNP-Man/mLucs prepared with different N/P ratios were measured using Cell Counting Kit-8 (CCK-8) in accordance with the instructions. A549 cells were seeded at a density of 1×10^4^ cells/well on 96-well plates, to which the prepared LNP-Man/mLucs were added. After 24 h of incubation, 10 μl of CCK-8 buffer was added into each well. After the following reaction at 37°C for 4 h, the absorbance was determined at 450 nm. Then, using the cells not exposed to LNP‐Man as control, the survival rate was 100%.

### Western Blot

After 24 h, the cells transfected with LNP-Man/mRNAs (Flu, Flu-Fe, or CD5-Flu-Fe) were collected and lysed in RIPA buffer supplemented with a PMSF protease inhibitor. The denatured protein in cells and the supernatants (only in the CD5-Flu-Fe group) were subjected to SDS‐polyacrylamide gel electrophoresis separation and then transferred onto a polyvinylidene fluoride (PVDF). The membrane was blocked for 1 h in 5% skimmed milk; then, the HA, M2e, and β-actin protein were respectively detected by anti-HA, anti-M2e, and anti-β-actin antibody (Sino Biological, Beijing, China). After horseradish peroxidase (HRP)–labeled secondary antibody staining, the protein bands were visualized by chemiluminescence.

### Vaccination and Protective Efficacy in Mice

Female C57BL/6 mice aged 6–8 weeks old were assigned randomly into seven groups (15 mice/group), including five immune groups (LNP-Man/Flu, LNP-Man/Flu-Fe, LNP-Man/CD5-Flu-Fe, LNP-Man/Flu-Fe+R848, and LNP-Man/CD5-Flu-Fe+R848) and two control groups (LNP-Man and PBS). Mice were vaccinated by intramuscular administration on the 0, 21st, and 42nd day. Mice in immune groups were immunized with 10 μg of LNP-Man/mRNA formulated with or without 3 μg of R848 (MCE, Monmouth Junction, NJ, United States). As far as control groups, one group received 3 μg of LNP-Man/R848 alone with no mRNA and the other group was treated with phosphate- buffered saline (PBS). On the 14th, 35th, and 56th days after the first immunization, blood was collected from the mouse orbital vein by a blood collection needle. The serum was stored in aliquots at –80°C for subsequent enzyme-linked immunosorbent assay (ELISA) assays so as to evaluate the level of vaccine‐induced humoral immune response. Two weeks after the third immunization, five mice were selected randomly from each group to isolate spleen lymphocytes and evaluate the level of the vaccine‐induced T-cell responses. The remaining ten mice of each group were challenged intranasally with the 1×LD_50_ (median lethal dose) A/chicken/Jilin/9/2004(H5N1) influenza virus and monitored daily for body weights and survival for 2 weeks after being challenged. On the 5th day postinfection (dpi), the lungs of five mice in each group were separated for hematoxylin and eosin (H&E) staining and cytokine/chemokine expression analyzing.

### Enzyme-Linked Immunosorbent Assay

For the detection of virus-specific antibodies in mouse serum, 96-well ELISA plates were coated overnight with 100 μl per well of 2 μg/ml M2e peptides (SLLTEVETPIRNEWGSRSNDSSD) or HA2 peptides (NAELLVL) in coating buffer (Solarbio, Beijing, China). After 3 washes in PBST (PBS with 0.1% Tween 20), plates were blocked using 300 μl of 3% BSA (bovine serum albumin) for 1 h at 37°C. The diluted serums were added to the plate for 1 h at 37°C. Plates were washed three times and incubated with HRP-conjugated anti-mouse IgG (1:5,000), IgG1 (1:5,000), and IgG2c (1:5,000) (SouthernBiotech, Birmingham, AL, United States). After washing three times, the TMB substrate was added to each well and incubated for 10 min. Then, the reaction was stopped by adding a stop solution. The optical density (OD) was read at 450 nm by a microplate reader (Molecular Devices, San Jose, CA, USA). Cytokines in serum, such as IL-4, IL-2, and IFN-γ, were detected by using the corresponding ELISA kits (Thermo Fisher Scientific, Waltham, MA, United States).

### Flow Cytometer

Mouse spleens were harvested on the 56th day after the first vaccination, and single-cell suspensions were prepared by processing them through a 70 μm cell strainer ( BD Biosciences, San Jose, CA, USA). Red blood cells presenting in the splenocyte suspensions were lysed using Red Blood Cell Lysis Buffer (Beyotime, Shanghai, China). The cells were inoculated into 96-well plates, and 20 μM antigenic peptides (M2e+HA2) were added to each well. After 1 h of incubation, we added the Brefeldin A Solution (1X) to each well and incubated them for another 5 h. Then, we washed the cells once with PBS buffer, added FITC anti-mouse CD3ϵ, PE/Cy7 anti-mouse CD4, and the APC anti-mouse CD8a antibody simultaneously (BioLegend, San Diego, CA, United States). Next, we incubated them at 4°C for 45 min and washed them with PBS twice, after which, we added the Cytofix/Cytoperm™ Solution and incubated them at 4°C away from light for another 20 min. Then, we washed the cells twice by using the BD Perm/Wash™ Buffer (BD, San Jose, CA, United States) and divided each tube of cells equally into 3 tubes; next, we added the PE anti-mouse IL-2, IL-4, or IFN-γ antibody, respectively, and incubated them for 1 h at 4°C away from light. After being washed twice, the stained cells were put into flow cytometry (Beckman Coulter, Miami, FL, United States) for detection.

### Real-Time RT-PCR for Viral Load and Cytokine Gene Expression

Total RNA was extracted from 140 µl of lung homogenates using a virus RNA isolation kit (Qiagen, Hilden, Germany). Using gene-specific primers and probes (**Supplementary Table 1**), gene expression levels were detected by real-time RT-PCR performed on a 7500 Real‐Time PCR System (Thermo Fisher Scientific, Waltham, MA, United States). The expression of house-keeping gene β-actin was quantified in parallel for RNA normalization, and the relative expression of the target genes was calculated by the ΔΔ Ct method.

### Histological Examination of Lung Tissue

For histological analysis, the right side of lung fixed in 4% formalin was sent to the Wuhan Servicebio Technology CO., LTD, Wuhan, China for H&E staining and analyzing. The main manifestations of lung pathology were alveolar wall thickening, inflammatory cell infiltration, and hemorrhage. The degree of tissue lesions was divided into five grades: the score of no or very few lesions were 0; mild or a small number of lesions were 1; moderate lesions were 2; severe or multiple lesions were 3; and severe or a large number of lesions were 4.

### Statistical Analyses

**Figure 1:**
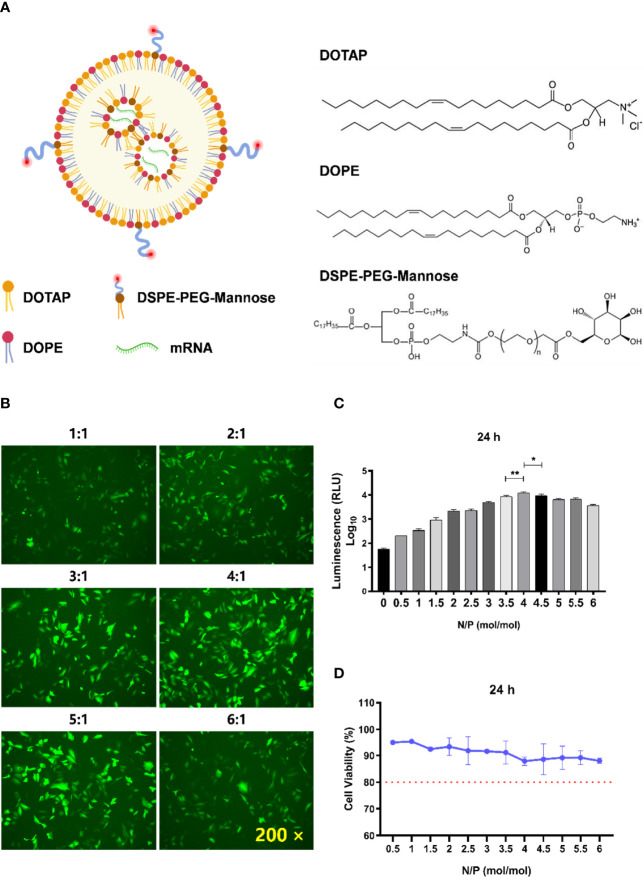
Determination of the optimal N/P molar ratio between LNP-Man and mRNA. **(A)** The schematic illustration of LNP-Man/mRNAs. **(B)** LNP-Man and mEGFP were mixed in accordance with the N/P molar ratio of 1:1, 2:1, 3:1, 4:1, 5:1, and 6:1. After transfecting A549 cells for 24 h, the results were observed by a fluorescence microscope. ×200 represents the magnification of the microscope. **(C)** Increasing the gradient number of N/P molar ratios between LNP-Man and mLuc, the results were read by a microplate reader after the transfection of A549 cells for approximately 24 h. Error bars indicate standard error of mean, n = 3 for each group. ^*^ p< 0.05, ^**^ p< 0.01, when compared with 3.5:1 and 4.5:1 by one-way ANOVA. **(D)** Toxic effects of LNP-Man/mLucs on A549 cells under different N/P molar ratios. The red dotted line represents 80% cell activity. Data were shown as mean ± SDs (n = 3).

Statistical analyses were performed with Excel and GraphPad Prism 8.0.2 software. The data presented in **Figures 1C**, **5**, **Figures 6A**, and **D–F** were analyzed for statistical significance using one-way ANOVA with multiple comparison. The data presented in **Figure 4** were analyzed for statistical significance using two-way ANOVA with multiple comparison. Survival analyses were performed using the log-rank (Mantel–Cox) test. The statistically significant difference in group means was indicated in figures as ^****^ p < 0.0001, ^***^ p < 0.001, ^**^ p < 0.01, ^*^ p < 0.05, or ^ns^ p >0.05.

## Results

### Determination of the Molar Ratio of N/P Between LNP-Man and mRNA

The schematic illustration of LNP-Man/mRNAs was shown in **Figure 1A**. The N/P molar ratio between LNP-Man and mRNA affects the transfection efficiency. In this study, green fluorescent protein (EGFP) and firefly luciferase (Luc) mRNA were selected as target genes. The transfection efficiency of LNP-Man/mRNA under different N/P molar ratios was determined according to the proportion and fluorescence value of fluorescent cells, respectively, so as to determine the optimal N/P molar ratio. Meanwhile, in order to determine the safe dose range, we also examined the cytotoxicity of different ratios of LNP-Man/mLuc on cells. The results of transfection of A549 cells with LNP-Man/mEGFP showed that when the N/P ratio was 4:1 and 5:1, the proportion of fluorescent cells in the field of view was higher than that of other gradient groups (**Figure 1B**). In order to determine the optimal ratio range more accurately, the transfection results of LNP-Man/mLuc indicated that when N/P was 4:1, the fluorescence value detected by the microplate reader was the highest, which was significantly higher than that of 3.5:1 and 4.5:1 groups (**Figure 1C**). Simultaneously, the cytotoxicity results of the test showed that the N/P molar ratio was transformed from 0.5:1 to 6:1, and the cell activity was higher than 80%, which was relatively safe (**Figure 1D**). Therefore, based on the meticulous observation, we confirmed that the optimal N/P molar ratio of LNP-Man to mRNA was 4:1, which can be used for subsequently *in vitro* transfection and *in vivo* immunization assays.

### Characterization of LNP-Man/mRNA

**Figure 2:**
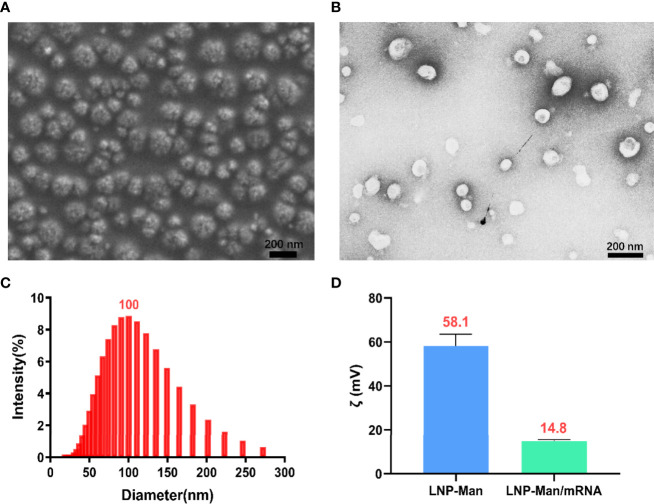
Characterization of LNP-Man/mRNA. **(A)** Scanning electron microscopy (SEM) and **(B)** transmission electron microscopy (TEM) images of LNP-Man mixed with mRNAs, as well as **(C)** particle diameter distribution diagram. **(D)** Zeta potential values of LNP-Man mixed with mRNA before and after. Data were shown as mean ± SDs (n = 3).

LNP-Man and mRNA were prepared according to the N/P molar ratio of 4:1, after which the particle form, diameter, and zeta potential were measured. The scanning electron microscopy (**Figure 2A**) and transmission electron microscopy (**Figure 2B**) showed that the nanoparticles were relatively equal-shaped spheres. The particle size distribution (**Figure 2C**) indicated that the particle diameter was approximately 100 nm. Finally, we measured the zeta potential of LNP-Man mixed with mRNA before and after. The results showed that the average zeta potential of LNP-Man was 58.1 mV, and the potential was decreased to 14.8 mV when mRNA was mixed with LNP-Man (**Figure 2D**). Due to analysis, the mRNA itself was negatively charged and could neutralize part of the positive charge of LNP-Man, whereas the final complex was still positively charged. Thus, during transfection, LNP-Man/mRNA can be electrostatically adsorbed to the negatively charged cell membrane, which facilitated the entry of mRNA into the cell.

### Expression Verification of Vaccine Antigen Genes

**Figure 3:**
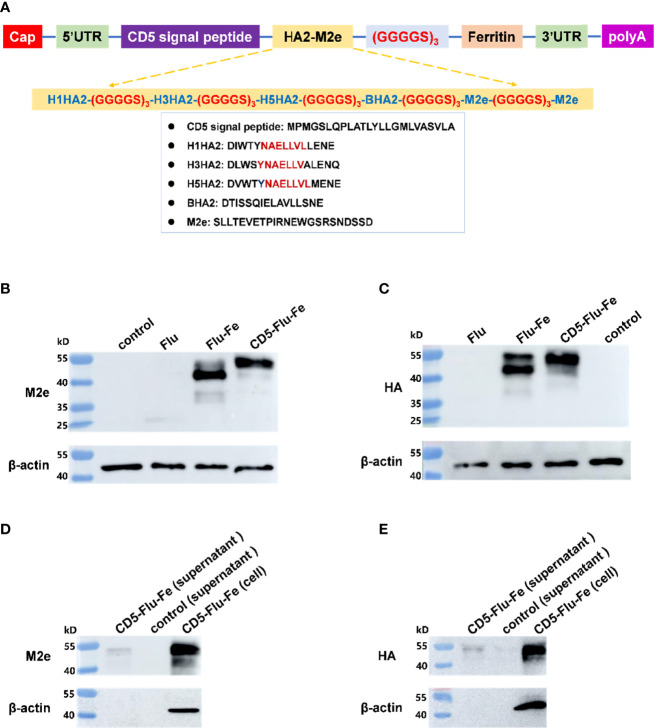
Expression verification of vaccine antigen genes. **(A)** The schematic illustration of antigen gene CD5-Flu-Fe. Cap, Cap structure of mRNA; UTR, non-translational region; HA2, the conserved antigen epitope in the stem region in influenza virus hemagglutinin gene; M2e, the conserved antigenic epitopes in influenza virus M gene; polyA, polyadenosyl nucleotide; GGGGS, a flexible linker. **(B)** Verification of intracellular M2e protein expression. **(C)** Verification of intracellular HA protein expression. **(D)** Detection of M2e antigen in the supernatant of CD5-Flu-Fe transfected cells. **(E)** Detection of HA antigen in the supernatant of CD5-Flu-Fe transfected cells.

The components of CD5-Flu-Fe and the amino acid sequences of CD5, HA2, and M2e genes were shown in **Figure 3A**. In order to verify the expression of antigen genes in mRNA vaccines, we prepared LNP-Man/Flu, LNP-Man/Flu-Fe, and LNP-Man/CD5-Flu-Fe at the N/P molar ratio of 4:1 respectively. The target proteins in cells of all groups and in the supernatant of the CD5-Flu-Fe group were detected by Western blot. The results of M2e antibody detection (**Figure 3B**) showed that M2e protein could be detected in all transfected cells. A shallow band appeared at 25 kD in Flu group, and the M2e expressive effect of the Flu-Fe and CD5-Flu-Fe group was better than that of Flu group. The results of HA antibody detection showed (**Figure 3C**) that the target protein in the Flu group was not detected, while the Flu-Fe and CD5-Flu-Fe groups could express the HA protein well. Meantime, M2e protein (**Figure 3D**) and HA protein (**Figure 3E**) were detected in the supernatant of the CD5-Flu-Fe group, and no β-actin protein was detected, indicating that the CD5 signal peptide could secrete the protein expressed in the cell to the extracellular. Overall, under the same dose of mRNA transfection, the introduction of a ferritin gene could better express the various components of the target antigen, so that the expressed antigens were more easily detected by antibodies.

### Immunization Route and mRNA Vaccines Induced Functional Antibody Responses in Mice

**Figure 4:**
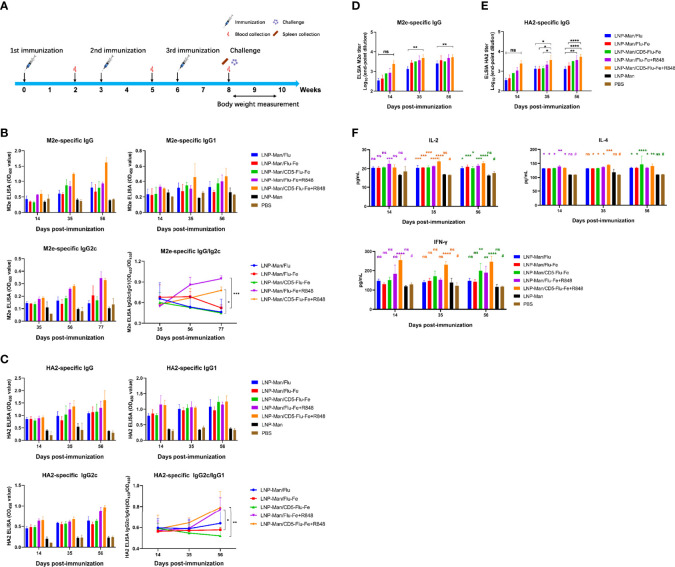
Immune response induced by mRNA vaccine in mice. **(A)** Immune procedure and experimental plan at different time points; **(B, C)** The secretion levels of specific antibodies IgG, IgG1, and IgG2c against M2e and HA2 in serum of mice on the 14th, 35th, and 56th days after immunization were detected by ELISA (n = 3). The variation trend of IgG2c/IgG1 in serum after immunization was detected by ELISA (n = 3). **(D, E)** The end-point titers of M2e- and HA2-specific IgG antibody in mice serum on the 14th, 35th, and 56th days after immunization (n = 3). **(F)** On the 14th, 35th, and 56th days after the first immunization, the secretion levels of IL-4, IL-2, and IFN-γ in mice serum were detected by the ELISA assay (n = 3). Data were shown as mean ± SDs and were analyzed by two-way ANOVA (^ns^ p > 0.05; ^*^p < 0.05; ^**^p < 0.01; ^***^p < 0.001; ^****^p < 0.0001). # represents the PBS group and is used as a reference group for comparison with the rest of the groups.

The immune procedure and the experimental plans at different time points are shown in **Figure 4A**. In order to detect the antibody level in mice after vaccination, the specific IgG/IgG1/IgG2c antibody against M2e (**Figure 4B**) and HA2 (**Figure 4C**) were determined. The results indicated that the specific antibodies were produced in all immune groups, and the OD_450_ values were gradually increased with the prolongation of immunization time. The results of IgG2c/IgG1 against M2e and HA2 indicated that the immune group with R848 adjuvant increased gradually with the prolongation of immune time gradually, while the immune group without R848 adjuvant had no significant change. Especially on the 56th day after immunization, the OD_450_ values of LNP-Man/Flu-Fe+R848 group was significantly higher than those of LNP-Man/Flu-Fe group, and the OD_450_ values of LNP-Man/CD5-Flu-Fe+R848 group were also significantly higher than those of the LNP-Man/CD5-Flu-Fe group. The results of IgG2c/IgG1 (25, 26) can show the differentiation direction of helper T cells. When the ratio increased, it indicated that the helper T cells tended to differentiate into T_h1_ phenotype. We also measured the titers of specific antibody IgG against M2e (**Figure 4D**) and HA2 (**Figure 4E**) in the mice serum on the 14th, 35th, and 56th days after immunization. The results indicated that the antibody titers in all immunized groups reached the highest level on the day 56th, which reached 1:6,400.

With the prolongation of immune time, the secretion levels of cytokines IL-4, IL-2 and IFN-γ in the five immune groups were significantly higher than those in the PBS group (**Figure 4F**), especially in the LNP-Man/CD5-Flu-Fe, LNP-Man/Flu-Fe+R848, and LNP-Man/CD5-Flu-Fe+R848 groups.

### mRNA Vaccines Induced Antigen-Specific T‐Cell Responses in the Spleen

**Figure 5:**
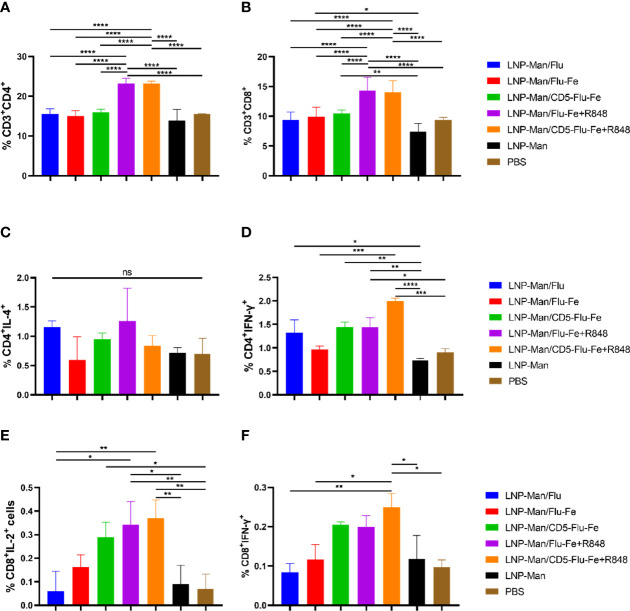
Antigen-specific T-cell responses in the spleen lymphocytes of C57BL/6 mice. On the 56th day after the first immunization, the proportion of antigen-specific CD4^+^ **(A)** or CD8^+^ **(B)** T cells was determined by flow cytometry analysis on splenocytes stimulated *in vitro* with M2e+HA2 peptides. Antigen-specific cytokine^+^ CD4^+^ T-cell subsets were identified as T_h1_ or T_h2_ depending on the intracellular cytokines, such as IL-4 **(C)** and IFN-γ **(D)**. Antigen-specific cytokine^+^ CD8^+^ T-cell subsets were identified on the basis of the combination of IL-2 **(E)** and IFN-γ **(F)**. Data were shown as mean ± SDs and were analyzed by ordinary one-way ANOVA multiple comparisons (n=3, ^ns^ p > 0.05; ^*^p < 0.05; ^**^p < 0.01; ^***^p < 0.001; ^****^p < 0.0001).

After the vaccination of C57BL/6 mice (n = 3 per group) with mRNA vaccines, we detected the cellular immune response. On the 56th day after immunization, lymphocytes from spleen were stimulated *in vitro* with M2e+HA2 peptides, and T-cell subsets and intracellular cytokines were measured by flow cytometry. All the five immune groups elicited antigen-specific CD4^+^ (**Figure 5A**) and CD8^+^ (**Figure 5B**) T cells, and the proportion of CD3^+^CD4^+^ or CD3^+^CD8^+^ cells in LNP-Man/Flu-Fe+R848 and LNP-Man/CD5-Flu-Fe+R848 immune groups were significantly higher than those in other groups.

To shed light on the functional characteristics of the helper T (T_h_) cells induced by the vaccines, we evaluated the capacity of the mRNA vaccines to shape the T_h_-cell potential to produce T_h1_-associated interferon (IFN)-γ (**Figure 5C**) and T_h2_-associated interleukin (IL)-4 (**Figure 5D**) upon *in vitro* stimulation. T_h_ cells from mice immunized with LNP-Man/Flu-Fe+R848 and LNP-Man/CD5-Flu-Fe+R848 showed a significant trend for a more pronounced T_h1_ polarization. In addition, the data of immune groups with an R848 adjuvant was increased in the antigen-specific CD8^+^ T-cell population with the administration of mRNA vaccines given by the contributions of IL-2^+^ (**Figure 5E**) and IFN-γ^+^ (**Figure 5F**) cells. The gating strategy and representative dot plots were presented in the Supplementary Material (**Supplementary Figure 1**).

### Protection Of rna Vaccines From 1×LD_50_ H5N1 Influenza Virus Challenge in Mice

On the 56th day after the first immunization, C57BL/6 mice were challenged intranasally with H5N1 influenza virus at the dose of 1×LD_50_. In order to evaluate the protective effect of the mRNA vaccines, the weight changes and survival rate of mice were monitored for 14 days. On the 5th day, five mice were randomly selected from each group. RNAs were extracted from their left lungs after being homogenated to determine viral loads and inflammatory cytokines/chemokines. The right lungs were fixed with 4% paraformaldehyde for pathological section preparation.

**Figure 6:**
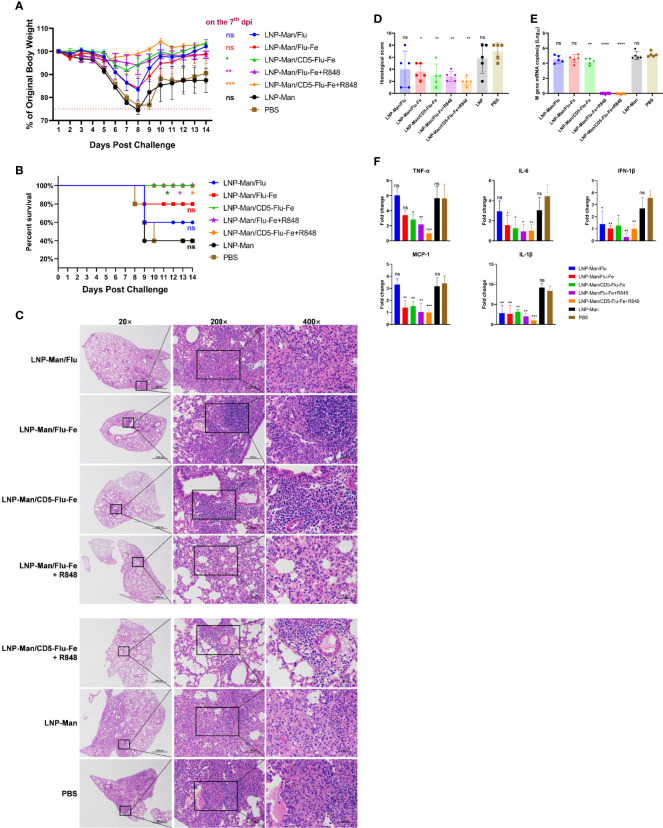
Analysis of protective effect of mRNA vaccines after challenge. After the third immunization, the C57BL/6 mice were challenged with H5N1 influenza virus at the dose of 1×LD_50_. The body weight **(A)** and survival rate **(B)** of mice were monitored for 14 days. Survival analyses were performed using the log-rank (Mantel–Cox) test. **(C)** Representative images of H&E-stained lung sections on the 5th day postinfection (dpi). n=5 for each group. **(D)** Results of histology scores. The viral loads **(E)** and the relative expression of cytokines/chemokines **(F)** in lung homogenate in each group on the 5th dpi were determined by real-time RT-PCR. Fold changes of the genes expression were relative to the lung samples in the LNP-Man/CD5-Flu-Fe+R848 group. Data were shown as mean ± SDs and were analyzed by one‐way ANOVA. Statistically significant differences are indicated in colors corresponding to the vaccine group (n=5, ^ns^ p > 0.05; ^*^p < 0.05; ^**^p < 0.01; ^***^p < 0.001; ^****^p < 0.0001 when compared with the PBS group).

The results of body weight monitoring (**Figure 6A**) showed that the body weight of mice in the LNP-Man/CD5-Flu-Fe group and the two groups with R848 adjuvant were relatively stable. The weight loss of control groups (LNP-Man and PBS) was the most obvious within 7 days after challenge. On the 7th dpi, the body weight of mice in the immune groups LNP-Man/CD5-Flu-Fe, LNP-Man/Flu-Fe+R848 and LNP-Man/CD5-Flu-Fe+R848 was significantly higher than that in the PBS group. The survival rate of mice (**Figure 6B**) indicated that unprotected mice died from the 8th day after challenge. Finally, the survival rate of immune groups LNP-Man/CD5-Flu-Fe, LNP-Man/Flu-Fe+R848 and LNP-Man/CD5-Flu-Fe+R848 was 100%, significantly higher than that of the LNP-Man and PBS groups (40%).

We amplified the severest lesions in the pathological sections (**Figure 6C**) by 200 times and 400 times, and invited pathologists for pathological analysis. In the PBS group and LNP-Man group, a large area of alveolar wall thickening, pulmonary substantiation, unclear alveolar structure, infiltration of lymphocytes together with granulocytes and diffuse infiltration of macrophages in the alveolar cavity were observed. To visually compare the differences in pathological damage among each group, we scored the pathological section results. The higher the score, the more serious the pathological damage (**Figure 6D**, **Supplementary Table 2**). The statistical results revealed that except for the LNP-Man/Flu group, the pathological damage degree of other immune groups was significantly lower than that of the PBS group.

In order to detect the change of viral load (**Figure 6E**) in the lungs, we measured the M gene copy number of the influenza virus. The real-time RT-PCR results indicated that the copy number of the LNP-Man/CD5-Flu-Fe group was significantly lower than that of the PBS group on the 5th day after the challenge. More importantly, there was no M gene detected in the two immune groups with an R848 adjuvant. Overall, it is the antigenic design of CD5-secreted signal peptide that matters in improving immunogenicity; meanwhile, the addition of R848 adjuvant significantly promoted the clearance of influenza virus in mice.

In order to analyze whether the vaccines can alleviate the inflammatory response in the lungs, we detected inflammatory-related cytokines/chemokines, including TNF-α, IL-6, IFN-1β, MCP-1, and IL-1β (**Figure 6F**). The relative real-time RT-PCR results indicated that only IFN-1β and IL-1β in the LNP-Man/Flu group were significantly lower than those in PBS group, while the rest of cytokines/chemokines in LNP-Man/Flu-Fe group were significantly lower than those in PBS group except TNF-α. More importantly, all cytokines/chemokines detected in the LNP-Man/CD5-Flu-Fe group and the two groups with R848 adjuvant were significantly lower than those in the PBS group.

## Discussion

The extracellular domain of influenza M2 protein (M2e) is highly conserved among influenza A viruses and considered a proper target for the development of universal influenza vaccine with broad-spectrum protection (27). In the previous studies, protective M2e antibodies had been induced in various ways including full-length protein with an adjuvant, as liposomes, tandem repeat formats (M2e–MAP), and recombinant expression with CD154 epitopes (28). As a hapten, HA2:90-105 is deficiently immunogenic, and thus, a carrier is crucial for improving its immunogenicity. The ferritin, a ubiquitous iron storage protein that self-assembles into nanoparticles (29), has been used to display exogenous peptides and demonstrated to be an ideal platform for presenting antigens. It is attractive for the development of broad-spectrum universal influenza vaccines to combine HA with M2e, and the same had been reported by earlier staff (30). According to a previous report, a trivalent chimeric norovirus P particle immunogen displaying influenza HA2 from subtypes H1, H3 and B have good immunogenicity and protective effects in mice (31). In this study, we attempt a new approach that makes a synthetic construct to link the conserved epitopes [H1/H3/H5/B-HA2(aa90~105)-M2e(24aa)] with ferritin to facilitate the formation of the larger immunogenic molecule so as to improve the immunogenicity of mRNA vaccines.

It was suggested by Beth C. Holbrook and colleagues that R848 conjugated to influenza virus induced a higher antibody response in neonates compared to the non-adjuvanted vaccine (32). A recent study had showed that prime-boost vaccination with a TLR7/8 agonist (R848)-conjugated influenza A virus vaccine elicited antibody responses to the highly conserved hemagglutinin stem and promoted rapid induction of virus neutralizing stem-specific antibodies following viral challenge (33). Thus, a hypothesis could be drawn that a robust humoral immune response would be induced by adding R848 adjuvant.

In this study, we evaluated the humoral immune response of five immune groups (LNP-Man/Flu, LNP-Man/Flu-Fe, LNP-Man/CD5-Flu-Fe, LNP-Man/Flu-Fe+R848, and LNP-Man/CD5-Flu-Fe+R848) in mice. All the sera were tested by the M2e and HA2 peptide ELISA. It was found that that all the immune groups produced specific antibodies against M2e and HA2, and the antibody levels increased in varied degrees with the prolongation of immunization time. Meanwhile, the antibody response level of the Flu monomer was lower than that of Flu-Fe structure after the booster. The same types of approach were followed by earlier staff (28). Increased level of IL-4 on the 56th day of all the five immune groups suggested the conversion of T_h2_ response. Additionally, increased level of IL-2 and IFN-γ on the 56th day of LNP-Man/CD5-Flu-Fe, LNP-Man/Flu-Fe+R848, and LNP-Man/CD5-Flu-Fe+R848 groups suggested the conversion of T_h1_ response (34).Additionally, the immune groups LNP-Man/Flu-Fe+R848 and LNP-Man/CD5-Flu-Fe+R848 promoted the production of the IgG2c antibody subtype, which was significantly higher than LNP-Man/Flu-Fe and LNP-Man/CD5-Flu-Fe, respectively. This indicated that the addition of R848 adjuvant to mRNA vaccines was conducive to promote the differentiation of T lymphocytes to the T_h1_ type. As far as we know, this is the first attempt to use R848 as an adjuvant in mRNA vaccines against influenza viruses.

Cellular immune response was monitored after immunization by flow cytometry. All the five immune groups elicited antigen-specific CD4^+^ and CD8^+^ T cells (35), the proportion of CD3^+^CD4^+^ or CD3^+^CD8^+^ cells in LNP-Man/Flu-Fe+R848 and LNP-Man/CD5-Flu-Fe+R848 immune groups was significantly higher than that in other groups. T_h_ cells from mice immunized with LNP-Man/Flu-Fe+R848 and LNP-Man/CD5-Flu-Fe+R848 showed a significant trend for a more pronounced T_h1_ polarization (36). The induction of antigen-specific CD4^+^ T cells was also found to be consistent, and in correlation with the observed IgG isotypes. In addition, the data of immune groups with R848 adjuvant showed an increase in the antigen-specific CD3^+^CD8^+^IL-2^+^ and CD3^+^CD8^+^IFN-γ^+^ T-cell populations (37).

During the challenge assay, we monitored the weight change and survival rate of mice, and analyzed the pathological lesions, viral load, and inflammatory-related cytokines/chemokines in lungs. It is suggested by a recent study that the host pro-inflammatory responses are one of the crucial contributing factors in the pathogenesis of H5N1 HPAI virus infection, and the fatal outcome could be mediated by a cytokine storm or hyper-acute dysregulation of pro-inflammatory cytokines (38). Pro-inflammatory cytokines have been observed in our study in control-infected groups, whereas the cytokine response was reduced in the LNP-Man/CD5-Flu-Fe, LNP-Man/Flu-Fe+R848, and LNP-Man/CD5-Flu-Fe+R848 groups as a protective response. Furthermore, the virus titers in LNP-Man/CD5-Flu-Fe, LNP-Man/Flu-Fe+R848, and LNP-Man/CD5-Flu-Fe+R848 groups are significantly reduced in the lungs after challenge.

Further studies are needed to assess the immune protection provided by these vaccines against other virus subtypes. The current research showed that M2e-HA2 recombinant protein had drastically reduced the pro-inflammatory cytokines and upregulated the innate immune system of chicken but failed to protect from a higher dose of HPAIV H5N1 challenge (28). Moreover, we need to further refine the vaccine antigen design and optimize the immunization dose and vaccination strategy. This study will be very useful in the future development of H5N1 avian influenza virus mRNA vaccines in terms of antigen designs and adjuvant selection.

## Conclusions

The protein expression efficiency is related to the design of antigen genes. Previous studies have shown that ferritin is a natural protein that can assemble itself to form nanoparticles, which is very suitable for antigen presentation and immune stimulation. Western blot results showed that ferritin did have a good antigen presentation ability and make each antigen component easier to be detected by antibodies. Meanwhile, the human CD5 signal peptide is considered to be an ideal signal peptide sequence for mediating the secretory expression of proteins. In the LNP-Man/CD5-Flu-Fe group, the survival rate of mice was 100%, the weight change was relatively stable, and the degree of lung injury and lung viral load were lower than those in LNP-Man/Flu-Fe group. Therefore, it can be concluded that the gene design pattern of secretory expression is conducive to improving the immune protection effect of mRNA vaccines.

In the challenge test, the survival rate of mice in the immune group containing an R848 adjuvant was 100%, and the weight change was relatively stable. Moreover, compared with other immune groups, the pathological damage of lung tissue was the slightest; the expression of inflammation-related cytokines/chemokines was the lowest; and the viral load was cleared. Accordingly, it can be concluded that the use of R848 adjuvant significantly improved the protective effect of mRNA vaccine.

In summary, from the above-mentioned study there are many factors that affect the effect of mRNA vaccines, including the selection of self-assembled polymer forms in an antigen design, the addition of signal peptides, the optimization of delivery vectors, and the use of vaccine adjuvants. As a result, in order to better protect humans and animals from influenza virus infection, researchers can optimize and innovate the mRNA vaccine platform from many perspectives.

## Data Availability Statement

The datasets presented in this study can be found in online repositories. The names of the repository/repositories and accession number(s) can be found in the article/**Supplementary Material**.

## Ethics Statement

The animal study was reviewed and approved by Changchun Veterinary Research Institute, Chinese Academy of Agricultural Sciences.

## Author Contributions

NJ, HL, MT, and XZ conceived and designed experiments. SX, TZ, BZ, TY, and NY performed gene cloning and expression. LC performed Western blot analysis. XZ, LC, SY, and SX performed vaccine formulation and mice vaccinations. WW, BZ, and ZX performed ELISAs. MT, XZ, and ZX performed the challenge of the mice with live H5N1. XZ, LC, and SY performed qRT-PCR. XZ, LC, and SY analyzed and interpreted the data. XZ, MT, HL, and NJ contributed to writing the manuscript. All authors contributed to the article and approved the submitted version.

## Conflict of Interest

The authors declare that the research was conducted in the absence of any commercial or financial relationships that could be construed as a potential conflict of interest.

## Publisher’s Note

All claims expressed in this article are solely those of the authors and do not necessarily represent those of their affiliated organizations, or those of the publisher, the editors and the reviewers. Any product that may be evaluated in this article, or claim that may be made by its manufacturer, is not guaranteed or endorsed by the publisher.

## Funding

This work was supported by National Major Scientific and Technological Special Project (2018ZX10102–001). The funders had no role in the study design, data collection and analysis, decision to publish, or preparation of the manuscript.
