# Supplementary material for: R848 Adjuvant Laden With Self-Assembled Nanoparticle-Based mRNA Vaccine Elicits Protective Immunity Against H5N1 in Mice
Source: Front Immunol. 2022 May 31;13:836274. doi: 10.3389/fimmu.2022.836274 (PMC9197463; doi:10.3389/fimmu.2022.836274)

Supplementary Material

**Supplementary Table 1. Sequences for primers used in real time** **RT-PCR assay**

| **Target** | **Gene** | **Forward primer (5’ to 3’)** | **Reverse Primer (5’ to 3’)** |
| --- | --- | --- | --- |
| Mouse | β-actin | ACGGCCAGGTCATCACTATTG | CAAGAAGGAAGGCTGGAAAAG |
| Mouse | TNF-α | ATAGCTCCCAGAAAAGCAAGC | CACCCCGAAGTTCAGTAGACA |
| Mouse | IL-6 | TGGAGTCACAGAAGGAGTGGCTAAG | TCTGACCACAGTGAGGAATGTCCAC |
| Mouse | IL-1β | GCCTTGGGCCTCAAAGGAAAGAATC | GGAAGACACAGATTCCATGGTGAAG |
| Mouse | MCP-1 | GGCTCAGCCAGATGCAGTTAA | CCTACTCATTGGGATCATCTTGCT |
| Mouse | IFN-β | TGGGAGATGTCCTCAACTGC | CCTGCAACCACCACTCATTC |
| H5N1 | M | GAGTCTTCTAACCGAGGTCGAAAC | TTAGTCAGAGGTGACAAGATTGGTC |
| H5N1 | M | Probe- (FAM)GCTTTGAGGGGGCCTGACGGG(TAMRA) | |

**Supplementary Table 2. Histopathological scoring criteria**

Each tissue section was carefully observed under a microscope and histological changes were recorded. The degree of tissue lesions was divided into five levels: the normal lung was scored as 0; mild or a small number of lesions were scored as 1; moderate lesions were scored as 2; severe or multiple lesions were scored as 3; very severe or a large number of lesions were scored as 4 (**a.** LNP-Man/Flu group; **b.** LNP-Man/Flu-Fe group; **c.** LNP-Man/CD5-Flu-Fe group; **d.** LNP-Man/Flu-Fe+R848 group; **e.** LNP-Man/CD5-Flu-Fe+R848 group; **f.** LNP-Man group; **g.** PBS group).

| **Lesion**  **Number** | **alveolar wall thickening** | **inflammatory cell infiltration** | **Total score** |
| --- | --- | --- | --- |
| a-1 | 1 | 4 | 5 |
| a-2 | 1 | 4 | 5 |
| a-3 | 0 | 1 | 1 |
| a-4 | 4 | 4 | 8 |
| a-5 | 0 | 1 | 1 |
| b-1 | 1 | 2 | 3 |
| b-2 | 1 | 4 | 5 |
| b-3 | 1 | 4 | 5 |
| b-4 | 1 | 1 | 2 |
| b-5 | 1 | 2 | 3 |
| c-1 | 0 | 1 | 1 |
| c-2 | 2 | 4 | 6 |
| c-3 | 1 | 2 | 3 |
| c-4 | 1 | 2 | 3 |
| c-5 | 0 | 2 | 2 |
| d-1 | 1 | 1 | 2 |
| d-2 | 1 | 1 | 2 |
| d-3 | 1 | 3 | 4 |
| d-4 | 2 | 1 | 3 |
| d-5 | 2 | 1 | 3 |
| e-1 | 1 | 2 | 3 |
| e-2 | 0 | 1 | 1 |
| e-3 | 0 | 2 | 2 |
| e-4 | 0 | 2 | 2 |
| e-5 | 0 | 2 | 2 |
| f-1 | 2 | 4 | 6 |
| f-2 | 1 | 1 | 2 |
| f-3 | 2 | 3 | 5 |
| f-4 | 4 | 4 | 8 |
| f-5 | 4 | 4 | 8 |
| g-1 | 2 | 4 | 6 |
| g-2 | 1 | 4 | 5 |
| g-3 | 4 | 4 | 8 |
| g-4 | 4 | 4 | 8 |
| g-5 | 4 | 4 | 8 |

**Supplementary Figure.1 The flow cytometry gating strategy and representative dot plots. (a)** Antigen-specific CD3^+^CD4^+^, CD3^+^CD4^+^IL-4^+^ and CD3^+^CD4^+^IFN-γ^+^ cells; **(b)** Antigen-specific CD3^+^CD8^+^, CD3^+^CD8^+^IL-2^+^ and CD3^+^CD8^+^IFN-γ^+^ cells.


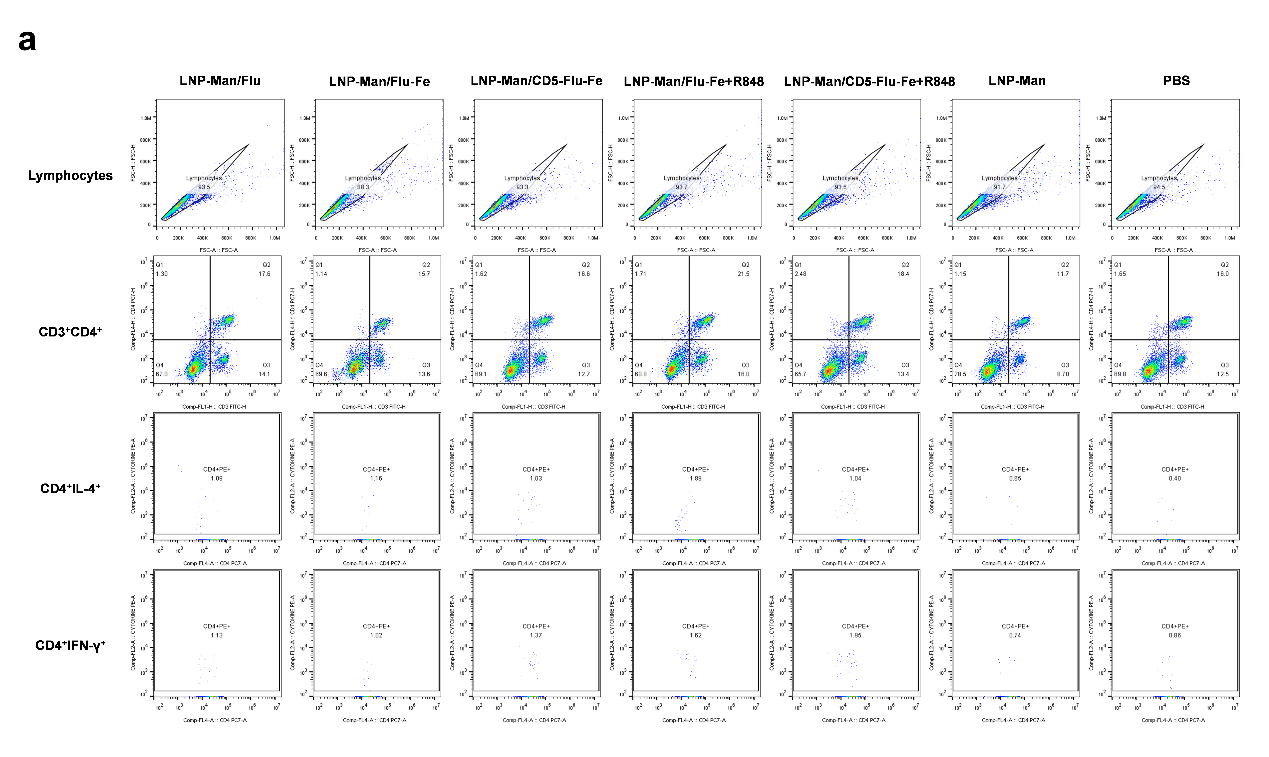


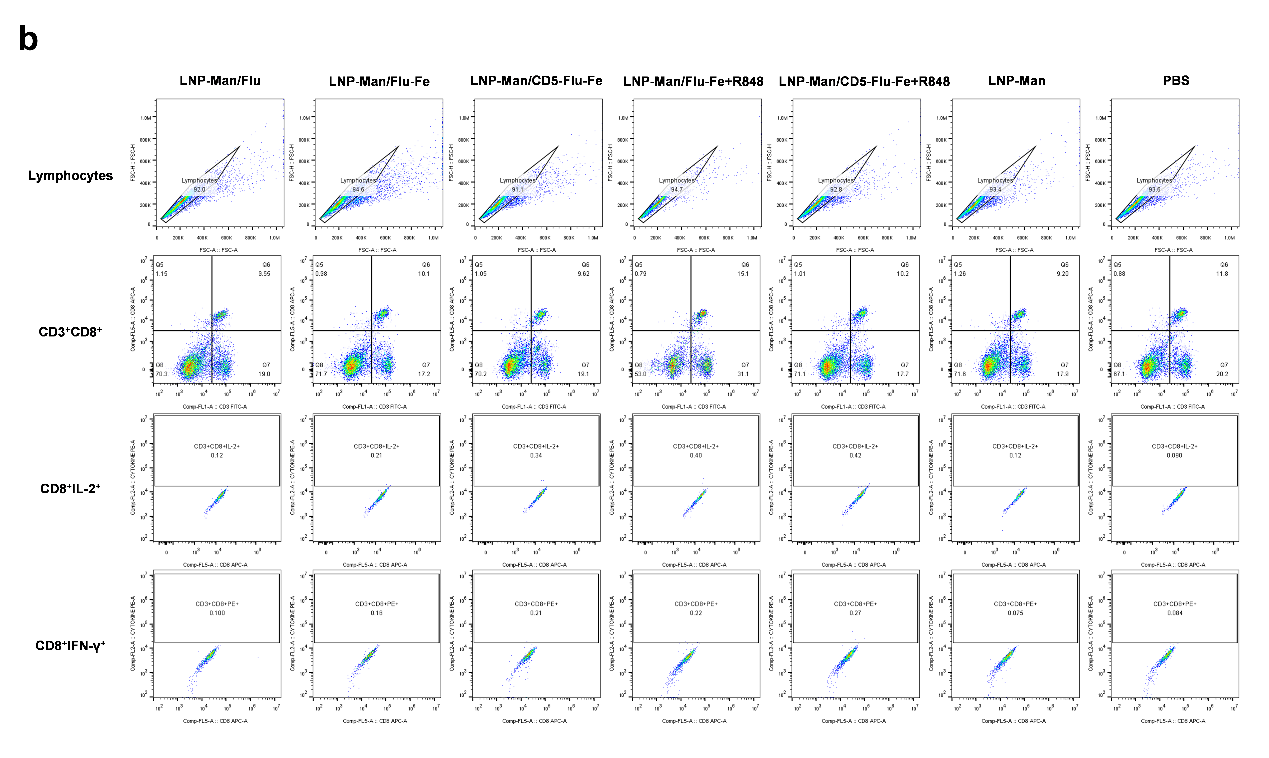

Supplement: Supplementary file 1 [file DataSheet_1.docx]
